# The racist origins, racialist connotations, and purity assumptions of the concept of “admixture” in human evolutionary genetics

**DOI:** 10.1093/genetics/iyad002

**Published:** 2023-01-27

**Authors:** Kostas Kampourakis, Erik L Peterson

**Affiliations:** Section of Biology and IUFE, University of Geneva, Pavillon Mail, 40 Boulevard du Pont-d’Arve, 1211 Geneva 4, Switzerland; Department of History, 202 ten Hoor Hall, Marrs Spring Rd., The University of Alabama, Tuscaloosa, AL 35487, USA

**Keywords:** admixture, population genetics, evolutionary genetics, racialism, racism, purity, race, ethnicity, ancestry

## Abstract

The concept of admixture is currently widely being used, both in population genetics research and in DNA ancestry testing discourse. It is assumed to describe the process of gene flow between 2 previously distinct populations that eventually become admixed because of this flow. The concept per se does not require pure or unadmixed populations; the changes are relative and what matters is the level of admixture before and after the event under consideration. However, in this paper, we argue that the concept of admixture as currently used assumes the existence of pure or unadmixed categories. These do not need to have actually existed but to be able to exist in principle. We argue that this is a problematic notion that accrues from the racialist origins of the term admixture, which, as a result, is based on assumptions about purity. We suggest that scientists should be very cautious in their use of this term, especially in science education and communication. We also suggest that the term admixture should be better replaced by terms denoting similarity rather than difference.

## Introduction

In several recent articles, concerns have been raised about the terminology used in the discourse related to ancestry and race. For instance, Lewis *et al*. (2022) have argued that researchers should refrain from using continental ancestry categories, which can be easily confused with racial groups, and embrace a multidimensional, continuous view of ancestry. Weasel (2022) has argued that current research questions and findings in comparative evolutionary genomics have drawn on and retained historical race biases from older human evolution research. Birney *et al*. (2021) have argued that geneticists need a more critical understanding of the societal impact of their work and of how it is communicated, in order to minimize or prevent misunderstandings. In the same spirit, in the present essay, we critically consider the use of the concept of “admixture” in human evolutionary genetics.

The concept of “admixture” is currently widely used, both in population genetics research and in DNA ancestry testing discourse. In population genetics, it is assumed to describe the process of gene flow between 2 previously distinct populations that eventually become admixed because of this gene flow. In DNA ancestry testing, it is assumed to describe the proportions of the different ancestries that an individual has. As used in population genetics, the concept of admixture per se does not require the existence of pure or unadmixed populations; the changes are relative and what matters is the level of admixture before and after the event under consideration. However, in this essay, we argue that when reference is made to individuals, the concept of admixture as currently used does implicitly assume the existence of pure or unadmixed categories. These categories do not need to correspond to groups that have actually existed, but to be able to exist or to have existed in principle. We argue that this is a problematic notion due to the racist origins of the term admixture, which, as a result, is based on assumptions about racial purity. Even though these racist assumptions either do not exist anymore or are less explicit, we argue that due to them, the concept of admixture as used today has racialist connotations—racialism being the idea that races are biologically real divisions of humans without any hierarchization.

We suggest that for these reasons scientists should be very cautious in their use of the term admixture, especially in science education and communication. We also suggest that the term admixture should be replaced by terms denoting similarity rather than difference. We propose the term “DNA sequence similarity” or simply “similarity” as an alternative.

## Racist origins of the concept of “admixture”

Human races viewed as distinct, pure, essential, and natural categories were socially constructed from the beginning of the early modern period (Graves and Goodman 2022). The notion that human races could be “admixed” likewise comes with substantial historical context. Historically, the concept of *admixture*, perhaps borrowed from 18th-century chemistry, appeared in the agricultural realm over the 1700s and was contrasted with the *purity* of essentially distinct breeds. In the 1780s, the Bakewell System of sheep breeding stressed “breeding in and in,” and, at times, the controversial notion of mating father to daughter or granddaughter and mother to son or grandson. James Anderson and William Marshall argued at the turn of the 19th century that breed must be composed of both “blood,” by which they meant some hereditary essence, and “soil,” by which they meant what we would call “environment” or perhaps “nurture” vs hardened “nature.” Blood and soil would need to be aligned from parent to child, “kind” to “kind,” in order to produce the purest breeds. But without some variation, presumably triggered by different environmental conditions and different “soils,” Anderson and Marshall expressed doubts whether there could be beneficial new traits that would be selectable to improve the breed (Wood and Orel 2001).

By the close of the 1700s, this discussion of breed in animals and plants had migrated to humans and discussions of race all across Europe and became much “harder” and more deterministic. With his “genetische Kraft” concept, leading sheep breeder Baron J. M. Ehrenfels stressed overall racial genetic/type stability in the face of individual variation (Poczai and Santiago-Blay 2021). Widely read French ethnographer Julian Joseph Virey agreed, mourning the degradation of original human stems (“genre”) through intermarriage. One could literally hear the degeneration as the “mother tongues” of each of the 5 pure human races mixed. This racial admixture (“les mélanges de races diverses”) signified society-wide corruption (Virey 1800).

In Scotland, preeminent anatomist Robert Knox insisted, “Men are of various Races; call them Species, if you will…. Now, the object of these lectures is to show that in human history race is everything” (1850, pp. 9–10). In Britain, Knox saw more races/species than Virey: “Three large bodies of men, of sufficient numerical strength to maintain, if not political power and unity, at least their integrity as a race distinct from others, in sufficient numbers to resist the aggressive action of the admixture of race by intermarriage…. [T]he Celtic, Saxon, and Belgian or Flemish” (1850, pp. 17–18). Human racial variation, in other words, was both cultural and biological and derived not from ordinary environmentally induced causes but from an admixture with the members of other races.

Over the 19th century, “admixture” carried an increasingly negative connotation. When scholars chose to use the term, they wanted to reinforce a lack of adherence to social codes about racial purity. In his 1843 work, “The Mulatto a Hybrid—Probable Extermination of the Two Races if the Whites and Blacks are Allowed to Intermarry,” influential American physician Josiah Clark Nott drew a hard equivalency between race and species, especially between Whites/Caucasians and darker-skinned groups considered to be lesser in their very essence. Given his observations and the stories of greater morbidity and mortality of interracial individuals that he collected from other physicians, Nott opined that mating between Whites and Blacks produced unfit *hybrids*—literally the offspring of 2 different species. Any society that permitted interracial marriage, he insisted, would biologically degenerate and eventually collapse. Justice H. M. Somerville, from Nott's home state of Alabama, echoed these fears in a court case that upheld interracial marriage bans across the United States for the next 80 years. Such admixture violated “the highest interests of government and society,” proclaimed Somerville, and resulted in “a mongrel population and a degraded civilization” (Pace & Cox v State 188*2*).

Other American naturalists agreed with Nott and Knox. Even those like John Bachman, who disagreed that races were separate species, nevertheless found racial admixture between Whites and Blacks personally, socially, and scientifically “revolting”:

[T]he white race has not only everywhere established its superiority over the African, but has won its way to all manner of intercourse. … In regard to the admixture of a superior with an inferior race in America, which in almost every case results in degradation and crime, it should be discountenanced by every lover of virtue, of good order and of sound morality. (Bachman 1850, pp. 105–106)

Admixture, again, was a term describing sullying, corrupting, and “making the pure White race impure.”

Even Charles Darwin, advocate for abolitionism and common descent that he was, still left the taxonomic classification of human race ambiguous. When he explored the issue in *The Descent of Man*, Darwin downplayed the issue: “[I]t is almost a matter of indifference whether the so-called races of man … are ranked as species or sub-species; but the latter term appears the more appropriate.” Yet, it was difficult for him to avoid reifying the essential status of human races, noting that “their manner of formation is closely analogous to that of natural species” (Darwin 2004 [1879], p. 210).

Neither the global retreat of the slave trade nor the rise of Darwinism foreclosed upon this belief in discrete and determinate biological races with the accompanying fear of racial admixture. These developments did make this belief slightly more scientifically precise. Ethnologists such as Britain's John Crawfurd noted that “The union of closely allied species of the human race produces no appreciable change in the offspring. This applies to all the different races of the European family. The people of Italy have suffered no degradation from a large admixture of England….” (Crawfurd 1861, p. 356). “Commixture,” Crawfurd delineated as an incidental interracial mixture of much further apart races that, over a few generations, left little trace—unless “there be a fresh infusion of the blood” of 1 of the major types of humans (e.g. Caucasian, African, Mongoloid). Otherwise, swamping would occur and humans would revert to racial type (Crawfurd 1863, p. 209). For these sorts of late-19th-century life and social scientists, the assumption of pure racial categories was explicit and externally identifiable, tied to essences carried in the blood.

Francis Galton began to move the discussion of types away from obvious external characteristics and toward traits tied to less visible, perhaps even invisible, discrete biological packets (though the term “genes” would not be coined for a generation). Galton spearheaded multiple sets of experiments reinforcing that types breed true, reacting against his cousin Darwin's overly “soft,” gradual, and a mathematical concepts of heredity and variation. From breeding moths to gathering human measurements at English fairs, Galton became convinced that organisms “revert” or “regress” to the mean of their “type,” even if their parents deviated far from that type (Galton 1887, 1888). He absolutely meant for this natural law of heredity to apply in the case of humans (Galton 1894). As he revealed in popular articles such as “Africa for the Chinese,” Galton saw the main benefit of his work in the life sciences in the way it would alter the human population of the entire globe. British gentlemen scientists would purify the human stock by preventing the weak and degenerated races from breeding at all, colonizing and displacing them from prime real estate if necessary, and by keeping Whites as racially healthy and free of admixture as possible (Galton 1873). Not surprisingly, he considered admixture as a process that destroyed purity:

Of course there are different degrees of stability. If the same structural form recurs in successively descending generations, its stability must be great, otherwise it could not have withstood the effects of the admixture of equal doses of alien elements in successive generations. Such a form well deserves to be called typical. A breeder would always be able to establish it. It tends of itself to become a new and stable variety; therefore all the breeder has to attend to is to give fair play to its tendency, by weeding out from among its offspring such reversions to other forms as may crop up from time to time, and by preserving the breed from rival admixtures until it has become confirmed, and adapted in every minute particular to its surroundings. (Galton 1889, p. 25)

Until the end of the 19th century, admixture had been considered as something detrimental to racial purity, and it was a term used by naturalists who were explicitly racist, at least in the sense that they considered some human groups as inferior to others (Blum 2002), most often to White Europeans and Americans.

## Retained racialist connotations in the era of Genetics

One of Galton’s young admirers, William Bateson, was the person who coined the term Genetics and promoted the work of Mendel, and the concept of Mendelism, to the English-speaking world. While he avoided taking a position on Galton’s eugenic plans for humanity, he strenuously defended the notion that biological “types,” including racial types, must be identified by particular genes. Genetics merely meant transferring traditional notions of types or kinds onto chromosomes and, eventually, DNA (Peterson 2008).

Bateson's ally Wilhelm Johannsen, who coined the term “gene” in 1909, also began to solidify the perspectives of Galton and Bateson in “Om Frøhviden og dens Udvikling hos Byg” (“Of the endosperm and its development in barley” 1884), which initiated Johannsen's famous pursuit of “pure lines.” For Johannsen, the purity of an organism was not a function of the biologist's average of all organisms deemed the same thing, but an actual underlying pure essence. Years later, Johannsen named this pure essence the “genotype” and considered it only partially connected to the variable phenotypic expression of the type. Johannsen dedicated his lauded pure-lines work through the turn of the 20th century to ensure there was no admixture in breeding lines (Roll-Hansen 2009). Variation, in that case, would be merely environmental. “Blood” would be stable, to use Anderson and Marshall's old terms, even if “soil” altered the phenotype slightly and in a nonheritable way.

Not surprisingly, then, by the 20th century, biologists of all stripes had defended the notion that races could be pure, essential categories represented by genes. Admixture of races meant that these races stood in danger of being made impure. In the decades leading to WWII, geneticists gradually deemphasized the *ranking* of races but retained their essential *reality* as pure categories of analysis. Well-known maize botanist and eugenicist George Harrison Shull, for example, vocally advocated outlawing interracial marriage as an “admixture” of the White race well into the 1930s. Ironically, Shull's antiadmixture advocacy largely contradicted the findings in his own groundbreaking maize genetics research (Shull 1905, 1909; Micklos 2009).

Anthropologists and geneticists reacted against such racialist and eugenicist connotations early on. Among others, anthropologist Franz Boas, cultural anthropologist Alfred Kroeber, physical anthropologist Sherwood Washburn, and geneticist Theodosius Dobzhansky attempted to discredit such notions. The scientific advancements of the 1930s and the 1940s (what came to be called the Modern Evolutionary Synthesis) rejected the typological thinking that was a prerequisite for racialist and eugenic theorizing. Rather than being considered as types, races were described as “biogeographically distinctive populations,” which contained a lot of genetic diversity, but which could also interbreed with one another (Jackson and Depew 2017).

Subsequent developments in the life sciences after WWII have overall rejected any notion of biological race as a legitimate scientific concept. However, the idea that genetically distinct human populations exist has persisted in many corners of biology, anthropology, and medicine. Even some scientific champions of anti*racism* continued to support *racialism*, at least implicitly. For example, in 1970, Theodosius Dobzhansky wrote that, whereas races were in general allopatric, humans were an exception: “Civilization created a variety of social forces that make possible, at least for a time, the sympatric coexistence of human races.” He also noted that a race “…consists of individuals who differ genetically among themselves. It is important to realize that similar genetic elements are involved in individual and in race differences. … Blue-eyed individuals are not a race distinct from brown-eyed ones, yet eye color is one of the trait distinguishing races.” Yet, on the other hand: “The obvious fact is, however, that members of the same species who inhabit different parts of the world are often visibly and genetically different. This, in the simplest terms possible, is what race is as a biological phenomenon” (Dobzhansky 1970, pp. 267–269).

Some geneticists today maintain Dobzhansky's racialist line of thought, despite their explicit rejection of race. For instance, as recently as 2018, David Reich argued: “Today, many people assume that humans can be grouped biologically into ‘primeval’ groups, corresponding to our notion of ‘races,’ whose origins are populations that separated tens of thousands of years ago. But this long-held view about ‘race’ has just in the last few years been proven wrong….” However, he also insinuated throughout his book that there exist distinct human populations: “The right way to deal with the inevitable discovery of substantial differences across populations is to realize that their existence should not affect the way we conduct ourselves.” (Reich 2018, p. xxiv). His perception of the substantial differences across populations led him to write that the divergence between West African and Europeans has not occurred for too long, adding that, “In light of this the lack of infertility in hybrids of present-day humans may no longer seem so surprising” (Reich 2018, pp. 49–50).

Writing about “hybrids of modern humans” might be construed as a racist comment, or not so, if one accepts that we are all hybrids anyway. Indeed, this seems to mainly be the conceptualization upon which most modern geneticists rely. There are no “pure” human populations, all of them are admixed, and the aim of geneticists is to figure out the extent of this admixture. So far so good. But this still relies on a problematic assumption.

## The assumption for the existence of “unadmixed” or “pure” categories

The notion of admixed populations and individuals assumes the existence of “unadmixed” of “pure” categories. For a population or individual to be admixed, there must exist at least 2 clearly distinct categories that can be sufficiently measured in order for the levels of admixture to be estimated. At the population level, it is certainly possible to estimate the contributions of predefined, ancestral populations, to the genetic makeup of contemporary ones. Population geneticists can define operational categories in specific contexts of interest. For example, if we define “Neanderthal” to be all humans who lived in Western Eurasia 100,000 years ago, and “non-Neanderthal” to be all humans alive in Africa 100,000 years ago, we can unambiguously identify the set of people who are 100% non-Neanderthal and the set of people who are 100% Neanderthal. Similarly, if we are talking about recent admixture in African Americans, we might define as “European” everyone who lived physically in Europe 1,000 years ago and “African” everyone who lived physically in Africa 1,000 years ago. The critical issue here is that the definition of a population is operational and has a temporal aspect that depends on the respective context. By considering the definitions within a particular context, the assumptions and limitations of these definitions can be made clear.

**Fig. 1.**
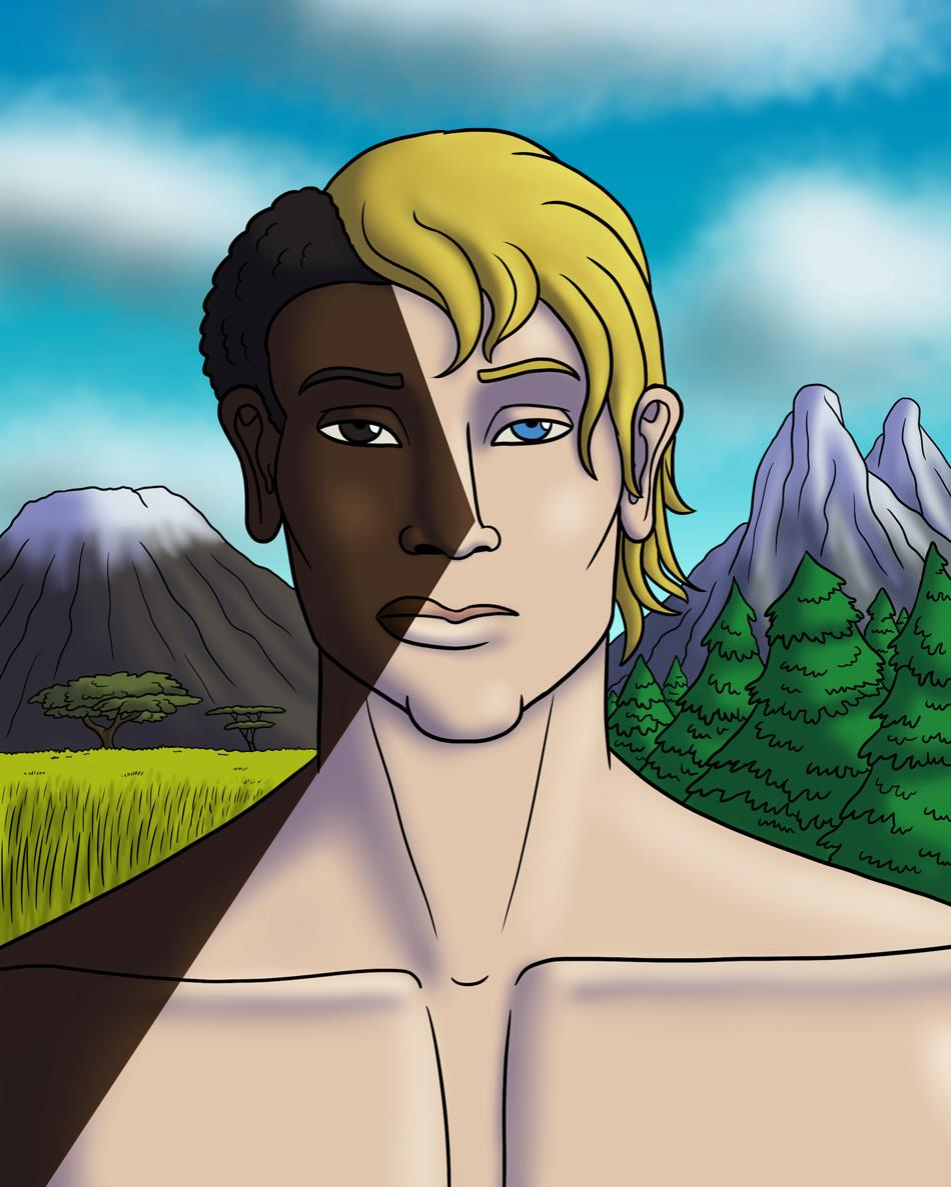
How individual admixture could be depicted, based on the assumption of “unadmixed” or “pure” and therefore clearly distinct categories. However, this creates the false impression that each individual's ancestry takes the form of a jigsaw puzzle that consists of different distinct categories. (Image credit: Brandon Pilcher, brandonpilchersart.com).

But this is not the case at all for discussions of admixture in individuals, for whom programs such as STRUCTURE and ADMIXTURE can provide admixture results, or who receive ancestry proportions/ethnicity estimates from the various DNA ancestry testing companies. Telling a person that they have, say, 66% European ancestry and 34% African ancestry, creates the false impression that each individual's ancestry takes the form of a jigsaw puzzle that consists of different distinct categories, as shown in Fig. 1. The idea of a person having multiple ancestries is certainly legitimate, as they might have for example approximately two-third of their recent ancestors (e.g. 5 out of 8 great-grandparents) from Europe and approximately one-third (e.g. 3 out of 8 great-grandparents) from Africa. However, receiving results that a person has 66% European ancestry and 34% African ancestry assumes the existence of 2 clearly distinct categories—in this case “African” and “European,” which an individual supposedly consists of. Let us consider why this is problematic.

A study, based on data from the database of the DNA testing company 23&Me, focused on the genetic ancestry of 5,269 self-reported African Americans, 8,663 Latinos, and 148,789 European Americans living across the United States. Using high-density SNP genotype data, they arrived at the genome-wide estimates presented in Table 1. These results indicate various levels of admixture of African, Native American, or European ancestry in today's African Americans, Native Americans, and European Americans (Bryc *et al.* 2015).

**Table 1.** Levels of genetic admixture among the customers of 23&Me.

Cohort	African ancestry (%)	Native American ancestry (%)	European ancestry (%)
African Americans	73.2	0.8	24.0
Latinos	6.2	18.0	65.1
European Americans	0.19	0.18	98.6

The first conclusion is clearly antiracialist in spirit: There are no “pure” cohorts, as all of them show signs of “admixture.” African Americans possess (on average) one-quarter European ancestry, and Latinos more than half of the same, whereas Europeans have some small amount of African ancestry. However, in order to be able to describe admixture as in Table 1, we need to be able to clearly distinguish between the 3 ancestry categories (African, Native American, and European). To do this, in turn, we need to be able to define what it means to have 100% African ancestry, 100% Native American ancestry, and 100% European ancestry. In other words, to describe any admixture, we need to be able to define what pure categories are being admixed. As the study discussed here relied on the 23&Me database, let us consider how this is done.

What they do is estimate what they call “Ancestry Composition report”, which shows the percentage of a person's DNA that comes from each of a total of 45 populations, consisting of over 14,437 people with known ancestry. When a segment of a person's DNA closely matches the DNA from 1 of the 45 populations, that ancestry is assigned to it. Then after estimating the ancestry for individual segments of the genome separately, these are added together to estimate a person's overall ancestry composition. This sounds reasonable until one considers the main assumption here: that the reference dataset of 14,437 people has “known ancestry.” These are people “… who were chosen generally to reflect populations that existed before transcontinental travel and migration were common (at least 500 years ago).” This is the first main assumption: there are particular populations that have existed before the colonization era, that have not undergone significant admixture and that have thus maintained their genetic variation (Assumption I). The second assumption is that the people included in the reference datasets “…have four grandparents all born in the same country—and the population of that country didn’t experience massive migration in the last few hundred years …”. So, having had your ancestors 2 generations back born in the same country makes a person's DNA representative of that country (Assumption II) (see 23&Me Ancestry Composition Guide; for a critical discussion of these assumptions, as well as of others about ancestry, see Kampourakis 2023).

This would mean, for instance, that a person who was born in Greece, a country that has not in recent times undergone significant admixture, at least compared with other European countries that had colonies during the last 500 years, and who has had all their grandparents born in Greece, would qualify as being included in the “Greek” reference group, as well as the “European” one. The reason for this is that both Assumption I and Assumption II are satisfied. However, both of these assumptions may be wrong.

Regarding Assumption I, there is ample archeological evidence that humans were migrating around the globe before the fifteenth century. Globalization, defined as a complex connectivity due to a dense network of intense interactions and interdependencies between disparate people brought together by the long-distance flow of goods, ideas, and people, is not a recent phenomenon. Of course, the pace and the extent in which this network has developed during the last 500 years or so is unparalleled. Yet, this does not mean that globalization did not occur in the past. In fact, some scholars have argued that throughout human history, there has been a single trend toward increasing globalization. Others have argued that globalization has occurred repeatedly in the past for certain periods of time. Whatever the case, the important point is that trends associated with globalization, such as those we observe today, can be found in earlier eras as well (Jennings 2017; Knappett 2017).

With respect to Assumption II, having had a person's grandparents born in the same country does not guarantee anything if there is no information about where their own parents and grandparents came from. To give an example, there are people who live in Greece today whose parents and grandparents were born in Greece, but whose great-grandparents might have been among the more than 1 million people who migrated to Greece from Asia Minor after the catastrophic events of 1922 and the population exchange of 1923. In particular, about 1,100,000 people moved to Greece from Asia Minor, and another 100,000 from Russia and Bulgaria, whereas about 380,000 people returned to Turkey (Clogg 2021, pp. 98–101). These, in turn, were people whose ancestors might have lived for generations in the areas from which they left and so their DNA variation would be more representative of that area, rather than from the area to which they eventually moved and raised their children.

Therefore, it seems that it is not possible to accurately define what it means to be 100% Greek, or anything else for that matter, because the assumptions necessary for this do not always stand. People have always been migrating and people from disparate places have always been mating and having offspring, and therefore, any assumption about unadmixed or relatively isolated (genetically) populations has to be considered with caution. In fact, and this brings us to a third issue, it is a common practice in studies of this kind to exclude people who were born and who live in a country if they did not have all their grandparents born there. This results in a kind of cherry-picking of data that eventually results in a circular logic that confirms the initial assumption. Excluding people from a study because they do not meet particular criteria and selecting only those who do, will in the end create a biased sample that will affirm the assumptions by which the sampling was made. Thus, the Greek reference group, for example, will be found to be distinct from other reference groups not because it actually is, but because of the sampling procedure that included in that group only those people who fulfilled the assumptions made (see DeSalle and Tattersall 2022; Kampourakis 2023).

If the concept of admixture relies on such problematic assumptions, might it then be replaced with something else?

## Replacing “admixture” with “similarity” in human genetics

In a recent paper, population geneticist Graham Coop (2022) has explained cogently that ancestry proportions are about similarity rather than ancestry (see also: Mathieson and Scally 2020). For instance, receiving results that someone like himself is 100% European means that, although we all share many genealogical ancestors, that person has many more paths back through their family tree to ancestors who are also ancestors many times over for other Europeans than they do with someone from Japan. As a result, that person shares slightly more genetic variants in common with another European than with a person from Japan. However, this does not in any way mean that all of that person's ancestors are European or that Europeans share some set of ancestors that people from other regions of the world such as Japan do not. Coop thus suggested that researchers need to stop using ancestry group labels such as Africans or Europeans and instead refer to similarity rather than ancestry (Coop 2022).

As a field we should move away from genetic ancestry labels and towards simple statements of genetic similarity: “This sample/haplotype is genetically similar to the XX sample set (in comparisons to YYY samples using ZZZ metric)” is much closer to how population genetic methods can be used to provide genetic sample descriptors. For example, “Graham is genetically similar to the GBR 1000 genome samples (on the first 10 principal components)” rather than “Graham has Northwestern European genetic ancestry”. The former sounds a little more awkward, but that awkwardness reflects the truth of how these labels work and comes with many fewer built-in assumptions and pitfalls.

Coop has thus provided us with a way that being 100% European or 100% African is meaningful: it reflects a person's similarity in terms of DNA markers with a particular reference group.

We therefore suggest that the concept of admixture should be replaced by another concept that refers to similarity. “Admixture” has always been about difference: about 2 entities that are so different that remain distinct even after they are put together. It is no coincidence that the concept of admixture was and is used in chemistry, often defined as the outcome of bringing together 2 or more substances that do not chemically react with one another. In the context of genetics, in order to study admixture in individuals, we have to define the assumed distinct categories and then measure the proportion of each one of them found in each individual. This perspective covers the problems discussed in the previous section. But if we turn this around, we can describe the same situation in terms of similarity. Let us see how this might be done.

Let us return to the example of a person who has, under current terms, mixed ancestry, for example, 66% European ancestry and 34% African ancestry. As Coop explains, this does not mean that two-third of this person's ancestors were European and one-third were African. Rather it means that for two-third of that person's DNA markers tested, more similarities can be found with the reference group described as European than with any other reference group we are using. Similarly, it also means that for one-third of that person's DNA markers tested, it is akin to finding more similarities with the reference group described as African than with any other reference group we are using. This, in turn, means that this person is more likely to have more common ancestors with the people in the former group than with the people in the latter group, and even less with the people in the other reference groups used in the study.

The same could be done for populations. Rather than saying that a population has European and African ancestry, we could say that this population exhibits a statistically estimated similarity to particular reference groups. Furthermore, this has the additional advantage of refraining from using ethnic denominations, which, in turn, risks the reification of ethnic categories such as Mycenaeans, Celts, and Longobards. Most importantly, instead of describing which distinct ancestries a population has, and how heterogeneous its genetic constitution is, we can rather focus on how similar it is to one or the other reference group. We acknowledge, however, that the concept of admixture properly used, with temporal references as described in the previous section, is less problematic than the concept of admixed individuals, or individuals with admixed ancestry.

For these reasons, we suggest replacing the concept of “admixture” in human population genetics and human ancestry testing with the concept of “DNA sequence similarity,” or simply “similarity” between a population or an individual, and a well-defined reference group. Therefore, instead of saying that an individual, like that shown in Fig. 1, has 66% European ancestry and 34% African ancestry, we could rather state that an estimated 66% of the DNA sequence shows higher sequence similarity with the European reference panel and an estimated 34% shows higher sequence similarity with the African reference panel—which, in turn, obliges the researchers and the DNA testing companies to clearly describe what these reference groups represent (Actually, we would also argue that continental, racial, or ethnic group categories should not be used at all, because it is easy to confuse them with the respective social groups, and because of the heterogeneity within continents and countries, the socially created boundaries between continents and countries, and the erroneous mapping of races onto continents and ethnic groups on countries, which often happen implicitly. We provisionally argue for the use of geographical denominators instead, but whether these should rely on the place of a person’s birth or where they live is a topic that merits its own discussion in another article.). We believe that such a practice would avoid the problems described in the present essay, distance modern research from the racist attitudes and practices of its past, and motivate more of us to place similarities among humans into sharper focus than differences.

## Data Availability

There are no data available related to this manuscript.
